# Transverse maxillary deficiency: treatment alternatives in face of early skeletal maturation

**DOI:** 10.1590/2177-6709.25.1.070-079.bbo

**Published:** 2020

**Authors:** Marcela Cristina Damião Andrucioli, Mírian Aiko Nakane Matsumoto

**Affiliations:** 1 Universidade de São Paulo, Faculdade de Odontologia de Ribeirão Preto, Curso de Especialização em Ortodontia (Ribeirão Preto / SP, Brasil).; 2 Universidade de São Paulo, Faculdade de Odontologia de Ribeirão Preto (Ribeirão Preto/SP, Brasil).

**Keywords:** Malocclusion, Palatal expansion technique, Ossification

## Abstract

Transverse deficiencies should be a priority in orthodontic treatment, and should be corrected as soon as diagnosed, to restore the correct transverse relationship between maxilla and mandible and, consequently, normal maxillary growth. Corrections may be performed at the skeletal level, by opening the midpalatal suture, or by dentoalveolar expansion. The choice of a treatment alternative depends on certain factors, such as age, sex, degree of maxillary hypoplasia and maturation of the midpalatal suture. Thus, the present study discusses different treatment approaches to correct maxillary hypoplasia in patients with advanced skeletal maturation.

## RESUMO

Os problemas transversais devem ser priorizados no tratamento ortodôntico e corrigidos assim que diagnosticados, para restituir a correta relação transversal entre maxila e mandíbula e, consequentemente, restabelecer o crescimento maxilar normal. A correção pode ser realizada em nível esquelético, por meio da abertura da sutura palatina mediana (SPM), ou por expansão dentoalveolar. A opção de tratamento depende de alguns fatores como idade, sexo, grau de hipoplasia da maxila e a maturação da SPM. Assim, o objetivo do presente trabalho foi discutir as diferentes abordagens terapêuticas para correção da hipoplasia maxilar em pacientes com maturação esquelética avançada.

## INTRODUCTION

Transverse deficiency,^1^ or maxillary hypoplasia,^2^ is one of the most detrimental problems to facial growth and to the integrity of the dentoalveolar structures. Therefore, it should be corrected as soon as diagnosed, to reestablish a normal transverse skeletal relationship between basal bones, fundamental to achieving a satisfactory and stable occlusion. It is usually characterized by posterior crossbite that may be unilateral or bilateral, total or partial, and may even not be present in cases with simultaneous mandibular arch constriction. Problems such as excessive vertical alveolar growth, crowding, deep and narrow palate with an intermolar distance of less than 31 mm, measured from the cervical margins, as well as large dark spaces in the buccal corridor, may be present, thus characterizing transverse maxillary deficiency as a syndrome.^1^ In addition, transverse maxillary deficiency may be associated with anteroposterior problems, and may be classified as real or relative. A Class II relationship may disguise a transversal involvement of the maxilla due to a posterior positioning of the mandibular arch, whereas in Class III, the anterior positioning of the mandible may accentuate maxillary deficiency or even project a non-existent deficiency.

The treatment of maxillary hypoplasia consists of rapid maxillary expansion (RME), which opens the midpalatal suture^3^ ^,^ ^4^ ^,^ ^5^ and should be conducted preferentially in growing patients, before suture ossification.^3^ ^,^ ^6^ RME before skeletal maturation peak has greater skeletal effects than when it is performed after growth peak,^7^ ^,^ ^8^ and is an unpredictable treatment for patients in the end of adolescence or early adulthood.^9^

According to several authors, the time during growth spurt or up to the age of 15 years is ideal for RME.^6^ ^,^ ^10^ Transverse growth of the palate due to osteogenic activity of the midpalatal suture persists up to the age of 16 years in girls and 18 years in boys.^10^ However, the fusion of the midpalatal suture varies greatly according to age and sex.^9^ ^,^ ^11^ ^,^ ^12^ ^,^ ^13^ The individual variability of midpalatal suture fusion should be understood to predict whether RME is a viable alternative in late adolescents or young adults.^9^

In patients in late adolescence or early adulthood, RME has limitations and complications, such as resistance to expansion, little or no opening of the midpalatal suture, predominance of dentoalveolar expansion instead of transversal gains of basal bone, excessive buccal tipping and extrusion of posterior teeth, gingival recession of supporting teeth, pain, palatal mucosa ulceration and even necrosis, as well as a high degree of relapse.^3^ ^,^ ^5^ ^,^ ^14^

The effect of RME on the palatal suture and periodontium depends on factors such as magnitude of the applied forces, treatment duration, frequency of activation and patient age. Alternatives to RME for patients with advanced skeletal maturation depend primarily on the degree of maxillary hypoplasia.

In cases with mild to moderate maxillary hypoplasia (of less than 5 mm, clinically measured in the region of the molars),^15^ in patients not growing, slow maxillary expansion may be indicated. In these cases, transverse maxillary remodeling may be achieved by the expansion of the alveolar processes and buccal tipping of crowns of the posterior teeth. These results may be achieved with the same appliances used in RME, such as Haas or Hyrax expanders, but activated at a lower frequency, or after the expansion of the maxillary arch and constriction of the mandibular arch by means of a fixed appliance.

In cases of severe maxillary hypoplasia (greater than 5 mm), several protocols for maxillary osteotomies have been developed to decrease the resistance to opening of the midpalatal suture, to separate the maxilla from its main cranial supports, and to obtain a permanent increase in maxillary width with minimal tooth inclination^16^. The two types of osteotomy more often found in the literature are the segmented Le Fort I maxillary osteotomy, which frees the maxilla from adjacent bones and defines segments to correct the transverse relationship during surgery (segmental maxillary expansion, SME)^17^, and partial maxillary osteotomy with the support of expanders to reduce resistance to expansion (surgically-assisted rapid maxillary expansion, SARME)^5^.

Recently, Lee et al.^4^ suggested a non-surgical approach to RME as an alternative to optimize the potential of skeletal expansion in patients with advanced skeletal maturation using mini-implants (miniscrew-assisted rapid palatal expansion, MARPE). This system applies forces to the miniscrews placed close to the midpalatal suture, differently from other techniques, which apply forces to the teeth or periodontium, therefore avoiding the need of osteotomies.^18^ ^,^ ^19^ MARPE is a less invasive option than SARME, has a skeletal effect, fewer dentoalveolar effects, no surgical risks and reasonably stable results, in addition to being more affordable financially.^20^ ^,^ ^21^

Thus, the objective of this study was to analyze and discuss different treatment approaches for the correction of maxillary deficiencies in patients with advanced skeletal maturation, and describe the treatment of a female patient (14 years and 4 months old) presenting Class II skeletal malocclusion, transverse maxillary hypoplasia and unilateral functional unilateral posterior crossbite - this case was submitted to the Brazilian Board of Orthodontics (BBO).

## CASE REPORT

**Figure 1:**
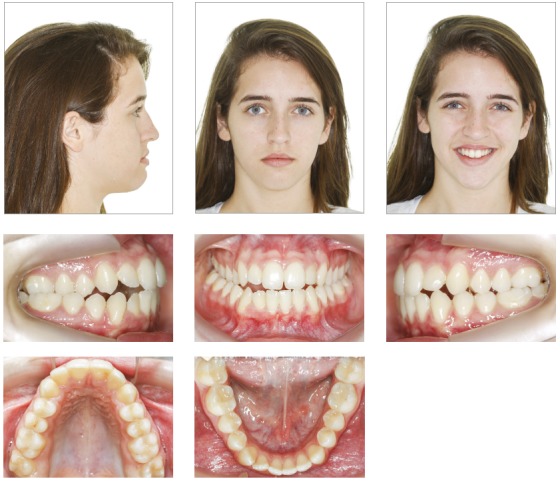
Initial facial and intraoral photographs.

**Figure 2:**
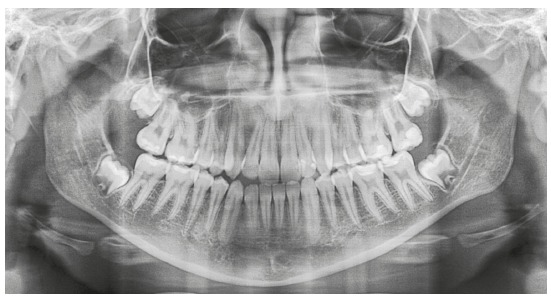
Initial panoramic radiograph.

A female patient (14 y 4 m) in good general health was referred to orthodontic treatment by her dentist. Her main complaint was functional: “bite instability”. She wanted to correct her “crooked bite”. Facial esthetics was not a concern for the patient or her mother. The frontal facial analysis revealed discrete mandibular asymmetry, thin upper lip and passive lip sealing. The analysis of her profile revealed an augmented lower third of the face and an anteroposterior deficiency of the middle third, as well as an evidently concave profile. A wide buccal corridor was found in the analysis of her smile. Clinical examination revealed a functional unilateral posterior crossbite, lower midline deviation to the right, and reduced overjet and overbite. Tooth #22 had no pulp vitality and there was an indication for endodontic treatment. Figures 1 and 2 show the initial facial and intraoral photographs and the panoramic radiograph.

The patient had severe mandibular deviation at mandible closure and absence of anterior and lateral guidance (protrusive and lateral excursions), but no temporomandibular joint signs or symptoms. The analysis of models of the maxillary arch revealed that is was constricted, with an intermolar distance of 30 mm, measured from the cervical margins.

**Figure 3:**
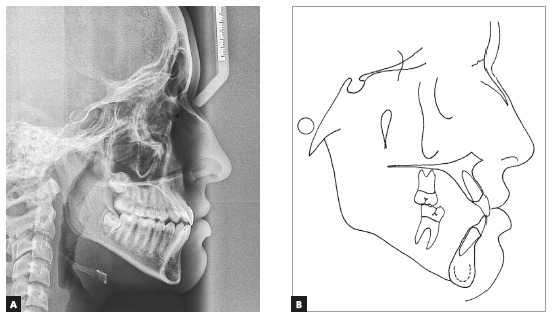
Initial cephalometric profile radiograph (A) and cephalometric tracing (B).

**Figure 4:**
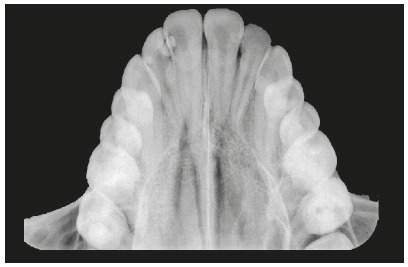
Initial occlusal radiograph.

The initial lateral cephalometric radiograph showed skeletal Class III malocclusion (ANB = -3˚, Wits = -7mm), due to greater maxillary retrusion (SNA = 73˚). The mandible was adequately positioned in the anteroposterior plane (facial angle = 88˚), and her bone profile was concave (facial angle = -6˚). The direction of facial growth was normal (Y-axis = 60˚), and the mandibular plane was larger than normal (SN.GoGn = 41˚, FMA = 30˚) (Fig. 3, Tab. 1). Maxillary incisors had a larger than normal axial inclination and were protruded (1.NA = 33˚, 1-NA = 10 mm), and the mandibular incisors were well positioned (IMPA = 92˚). The patient was in the descending phase of the pubertal growth spurt, in the CS5 stage.^7^ However, a radiolucent line was still visible in the midpalatal suture in the premaxilla on the occlusal radiograph (Fig 4).

## TREATMENT PLAN AND MECHANICS USED

**Figure 5:**
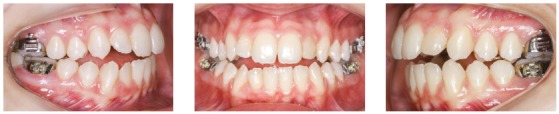
Intraoral photographs after slow maxillary expansion.

Initial treatment objectives included the correction of transverse maxillary hypoplasia with RME and improvement of smile esthetics, and preservation of the anteroposterior discrepancy and of the dental compensations. A Haas expander was used for RME, and the initial activation protocol was 4 activations on the first day (one full turn), followed by 2 daily activations for one week (1/2 a turn per day)^3^ and reassessment. As there was not interincisal diastema, which is a clinical sign of midpalatal suture opening, slow maxillary expansion was initiated with two weekly activations (½ a turn per week) because the patient had a mild maxillary hypoplasia, and posterior teeth had a normal buccal inclination. The appliance was activated until there was overcorrection, with the occlusal aspect of the lingual cusps of the maxillary molars occluding against the occlusal aspect of the buccal cusps of the mandibular molars. The correction of crossbite eliminated the mandibular deviation and the deviation of the mandibular midline, as seen on intraoral images obtained after slow maxillary expansion (Fig 5).

A fixed Edgewise appliance (Kirium, Abzil, 3M, São José do Rio Preto, Brazil) with a 0.022 x 0.028-in slot was used for maxillary alignment and leveling, together with 0.014 to 0.018-in NiTi archwires and 0.020-in and 0.019 x 0.025-in stainless steel archwires, expanded and with tightly attached omega loops. The open bite, which became larger after slow maxillary expansion, was corrected using posterior torques.

The mandibular arch was aligned and leveled using 0.014-in to 0.018-in round stainless steel archwires and a 0.019 x 0.025-in rectangular archwire as the initial archwire. Class II and III intermaxillary elastics were used to correct the anteroposterior relationship. Light 0.020-in archwires were used for finishing. The archwires were stabilized for 30 days, and a removable maxillary wraparound retainer and a lingual arch in 0.7-mm stainless steel wire bonded to canines were used until the appliance was removed. 

## TREATMENT RESULTS

**Figure 6:**
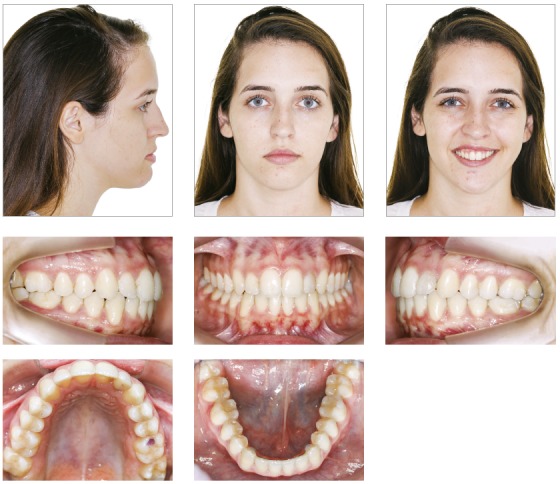
Post-treatment facial and intraoral photographs.

Treatment objectives were achieved. Smile esthetics improved because of a decrease of the buccal corridor. Facial profile improved because of the repositioning of the lower lip after a discrete clockwise rotation of the mandibular plane (Fig 6).

**Figure 7:**
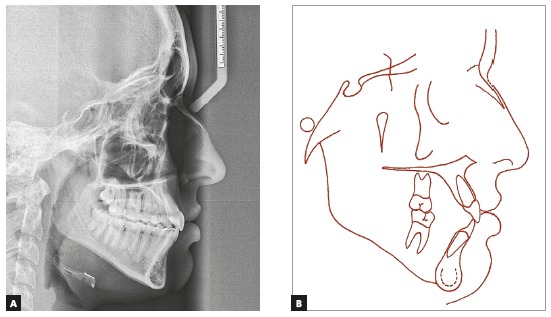
Post-treatment cephalometric profile radiograph (A) and cephalometric tracing (B).

The patient’s skeletal pattern was preserved, and there was a discrete improvement of the anteroposterior relationship of the basal bones (ANB = -2˚, Wits = -6 mm) and discrete increase of the mandibular plane inclination (SN.GoGn = 41.5˚). The axial inclination of maxillary incisors improved, but remained greater than normal, which compensated the skeletal Class III pattern. There was also a decrease of the 1.NB angle (28˚) (Fig 7, Table 1).

**Table 1:** Initial (A) and final (B) cephalometric values.

	Measurements		Normal	A	B	Dif. A/B
Skeletal pattern	SNA	(Steiner)	82°	73°	71.5°	1.5
	SNB	(Steiner)	80°	76°	73.5°	2.5
	ANB	(Steiner)	2°	-3°	-2°	1
	Wits	(Jacobson)	♀ 0 ± 2mm	-7mm	-6mm	1
			♂ 1 ± 2mm			
	Angle of convexity	(Downs)	0°	-6°	-6°	0
	Y-axis	(Downs)	59°	60°	60°	0
	Facial angle	(Downs)	87°	88°	88°	0
	SN.GoGn	(Steiner)	32°	41°	41.5°	0.5
	FMA	(Tweed)	25°	30°	29°	1
Dental pattern	IMPA	(Tweed)	90°	92°	92°	0
	1.NA (degrees)	(Steiner)	22°	33°	30.5°	2.5
	1-NA (mm)	(Steiner)	4 mm	10mm	11mm	1
	1.NB (degrees)	(Steiner)	25°	30°	28°	2
	1-NB (mm)	(Steiner)	4 mm	5mm	6mm	1
	- Interincisal angle	(Downs)	130°	120°	124°	4
	- Apo	(Steiner)	1 mm	6mm	6mm	0
Profile	Upper lip - S-line	(Steiner)	0	-3mm	-3mm	0
	Lower lip - S-line	(Steiner)	0	0.5mm	0mm	0.5

**Figure 8:**
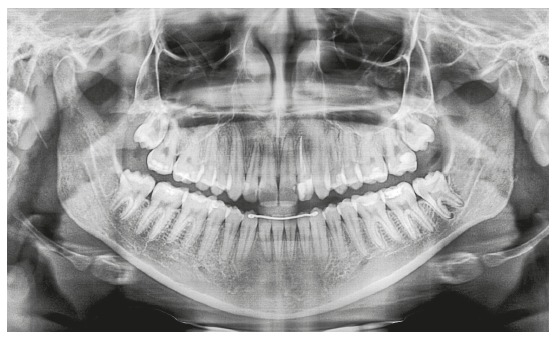
Panoramic radiograph after treatment.

Maxillary expansion corrected maxillary constriction, resulting in an intermolar distance of 33 mm, as well as eliminating mandibular deviation and, consequently, mandibular midline deviation. Ideal occlusion was achieved with correct canine and molar relations and normal overjet, overbite, dental intercuspation and disocclusion. Good root parallelism was achieved. Although indicated, third molars have not been extracted yet and remain under observation. Tooth #22 was treated endodontically before orthodontic treatment and, because of discoloration, would undergo esthetic restoration (Fig 8).

**Figure 9:**
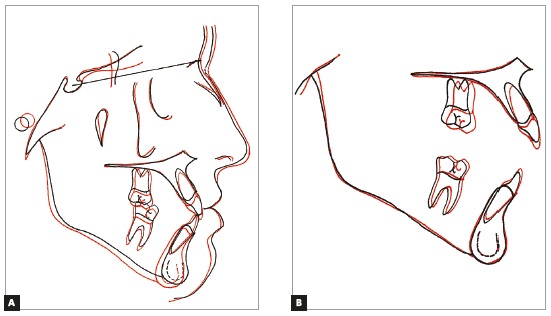
Initial (black) and post-treatment (red) total (A) and partial (B) superimpositions of cephalometric tracings.

Superimposition of cephalometric tracings revealed discrete facial growth during treatment and a small clockwise rotation of the mandible. The partial superimposition of the maxillary tracings revealed extrusion and mesial movement of molars and extrusion and uprighting of incisors. For the mandible, partial superimposition demonstrated the extrusion and mesial movement of molars and extrusion of incisors (Fig 9).

## DISCUSSION

The correct diagnosis of the severity of transverse deficiency and its skeletal and dentoalveolar components is fundamental for treatment success. The decision about the best treatment approach in the different cases of maxillary hypoplasia in patients with advance skeletal maturation depends on several factors, all of which should be analyzed together.

The fusion of the midpalatal suture varies greatly according to age and sex. Persson and Thilander^11^ reported on midpalatal suture fusion in patients aged 15 to 19 years. In contrast, there are reports of adult patients of different ages (27, 32, 54, 71 years) without signs of midpalatal suture fusion.^9^ ^,^ ^11^ ^,^ ^12^ ^,^ ^13^

As early as 1987, Bishara and Staley^22^ found that RME in late adolescence or early adulthood (young adults) might fail. Pain, ulcerations, palatal mucosa necrosis, accentuated buccal tipping of posterior teeth and gingival recession have been reported in the literature for cases in which RME failed^23^. 

Angelieri et al.^9^ evaluated the skeletal maturation of the midpalatal suture using cone beam CT (CBCT) to avoid failures in RME or surgical separation in older adolescents or young adults. In that study, they reported that 25% of the girls 11 to 14 years old and 57% of those 14 to 18 had midpalatal suture fusion in the palatal or maxillary bone.

In contrast, some studies found that the percentage of fusion^11^ ^,^ ^12^ ^,^ ^13^ has been classified as more important than the presence or absence of the midpalatal suture. According to Persson and Thilander,^11^ RME may be performed using conventional orthopedic forces applied to the sutures, with a fusion index below 5%. Indices below 5% have been described for patients aged 18-38 years^24^, 14 to 71 years^13^ and 18 to 63 years^12^. However, those studies did not explain why it is difficult to open the midpalatal suture in patients older than 25 years. Most of the resistance to midpalatal suture opening seems to be explained by the fusion of circummaxillary sutures^13^ ^,^ ^25^. In a recent study, Angelieri et al.^26^ found an association of the maturation stages of the zygomaticomaxillary suture and the response to RME followed by protraction.

In patients with advanced skeletal maturation, although the transverse skeletal gain is relatively small, dentoalveolar expansion may be an alternative to increasing palatal width and promoting posterior intercuspation at the end of a corrective orthodontic treatment, without, however, promoting the opening of the midpalatal suture, as radiographically evaluated.^14^ ^,^ ^27^

The present female patient, who was 14 years and 4 months old, had a maxillary transverse deficiency according to McNamara^1^, as the intermolar distance, measured from the cervical margins, was shorter than 31 mm. The initial activation protocol was RME, but, because of the patient's age and the maturation of the cervical vertebrae, as shown on the lateral cephalometric radiograph, the midpalatal suture might not open. RME may vary greatly with age, sex, bone characteristics and midpalatal suture ossification, and may be an unpredictable procedure at the end of adolescence.^9^ Cone beam CT (CBCT) scans were not requested, because, according to Isfeld et al.,^28^ their use as a diagnostic tool in daily clinical practice, as suggested by Angelieri et al.,^9^ is impractical due to costs and availability of time and resources. Moreover, there is no scientific evidence to justify their use in the accurate determination of midpalatal suture maturation. The comparison of histologic morphology and CBCT morphology is not compatible, as histologic findings are microscopic, whereas axial CBCT views of the sutures have a macroscopic or naked-eye scale. Therefore, the maturation stages demonstrated by Angelieri et al.^9^ using CBCT should be interpreted carefully, as part of an extended protocol for a subjective evaluation of midpalatal suture maturation. Because of that, other diagnostic criteria should be used for a subjective evaluation and a definition of the best clinical management. As soon as the absence of an interincisal diastema, the clinical sign of midpalatal suture opening, was detected, the activation protocol was changed to slow maxillary expansion, because maxillary hypoplasia was mild (30 mm) and there were no major complications.

Posterior dentoalveolar inclination should also be taken into account during palatal expansion planning^3^. Mild or moderate transverse maxillary discrepancies (up to 4 mm), with normal or reduced inclination of posterior teeth, such as in the case in this study, may be corrected with slow maxillary expansion^27^ ^,^ ^29^, as the correction will result from tipping of the lateral bone bases of the palate and of the posterior teeth, as well as from the remodeling of alveolar processes.

After the correction of crossbite, increased buccal tipping of the posterior teeth in the case reported here was corrected using buccal root torque, which also promoted the closure of the anterior open bite resulting from the expansion. Handelman^27^ also found an increase in buccal tipping of molars after RME.

An alternative to the treatment plan presented in this case may be MARPE^4^. According to Pereira et al.,^30^ palatal separation in MARPE is type I, a complete midpalatal suture separation from the anterior to the posterior nasal spine, whereas surgically-assisted RME is the incomplete separation of the midpalatal suture (type II separation). Moreover, Choi et al.^20^ found a success rate of 86.96% in preserving skeletal and dentoalveolar expansion and stability of periodontal structures during retention. The activation protocol recommended for MARPE is ¼ of a turn every day,^4^ so that the tissues adapt to the forces applied and patient discomfort is minimized, considering the increase in the rigidity of the midpalatal suture with age.^21^

## CONCLUSION

According to the literature and the clinical case described here, we concluded that slow expansion may be a treatment alternative to achieving a stable and functional occlusion in cases of early skeletal maturation of the midpalatal suture at the end of adolescence. 
